# ICS/LABA Combined With Subcutaneous Immunotherapy Modulates the Th17/Treg Imbalance in Asthmatic Children

**DOI:** 10.3389/fimmu.2022.779072

**Published:** 2022-03-10

**Authors:** Huan Dai, Rongying Zheng, Like Wang, Jinyi Wan, Yu Tong, Wei Zhao, Weixi Zhang

**Affiliations:** ^1^Department of Pediatric Allergy and Immunology, The Second Affiliated Hospital and Yuying Children’s Hospital of Wenzhou Medical University, Wenzhou, China; ^2^The Second Clinical Medical College, Wenzhou Medical University, Wenzhou, China

**Keywords:** asthma, inhaled glucocorticoids, SCIT (subcutaneous immunotherapy), Th17, Treg

## Abstract

**Rationale:**

The imbalance of T helper (Th17) cell and regulatory T (Treg) cell are involved in allergic asthma pathogenesis. We hypothesized that ICS/LABA could modulate the Th17/Treg imbalance and that subcutaneous immunotherapy (SCIT) could coordinate with ICS/LABA to rebalance the dysfunction of Th17/Treg.

**Methods:**

Thirty house dust mites (HDM) allergic asthmatic children and fifteen healthy control subjects were enrolled in this study. Fifteen asthmatic children were treated by ICS/LABA powder inhalation, while the other fifteen asthmatic children were treated by ICS/LABA powder inhalation combined with HDM-SCIT. Asthmatic subjects were followed up for 6 months, but 2 asthmatics treated with ICS/LABA were lost to follow-up. Flow cytometry was used to determine the proportions of Th17 and Treg in CD4^+^ T cells from peripheral blood mononuclear cells (PBMCs). Serum levels of IL-17A and IL-10 were assessed by ELISA.

**Result:**

ICS/LABA treatment significantly reduced the percentage of Th17 cells (1.252 ± 0.134% vs. 2.567 ± 0.386%), serum IL-17A (49.42 ± 2.643 pg/ml vs. 66.75 ± 3.442 pg/ml) and Th17/Treg ratio (0.194 ± 0.025 vs. 0.439 ± 0.072) compared to baseline (P<0.01). The ICS/LABA+HDM-SCIT treatment group showed similar reduction in the percentage of Th17 cells (1.11 ± 0.114% vs. 2.654 ± 0.276%), serum IL-17A (49.23 ± 2.131 pg/ml vs. 66.41 ± 2.616 pg/ml) and the Th17/Treg ratio (0.133 ± 0.015 vs. 0.4193 ± 0.050) (P<0.01). ICS/LABA+HDM-SCIT treatment group demonstrated elevated Treg percentages (8.483 ± 0.408% vs. 6.549 ± 0.299%) and serum IL-10 levels (127.4 ± 4.423 pg/ml vs. 93.15 ± 4.046 pg/ml), resulting in a lower Th17/Treg ratio than the ICS/LABA group.

**Conclusion:**

ICS/LABA treatment regulates Th17/Treg imbalance mainly by mitigating Th17-induced inflammation in asthma patients. The addition of SCIT further enhanced such effect by upregulating Treg cells.

## Introduction

Asthma is a common chronic airway disease in children, and is characterized by airway inflammation, airway hyperresponsiveness (AHR), reversible airflow obstruction and airway remodeling. Allergy is mainly responsible for asthma, in light of allergen exposure and sensitization involved in the occurrence and development of asthma. Allergen stimulation causes the recruitment of inflammatory cells including eosinophils, mast cells, and T lymphocytes, leading to inflammatory responses in asthmatics (1).

In the long-term exploration of the mechanism and treatment of asthma, glucocorticoids (GCs) have been widely acknowledged as the core therapy for asthma. GCs can reduce airway inflammation and restrain AHR, thereby improving the severity of symptoms and lung function clinically (2, 3). In terms of mechanism, GCs have direct effects on asthma-related inflammatory cells such as T-lymphocytes, eosinophils, mast cells and dendritic cells (4). Inhaled GCs (ICS) as single drugs or combined with long-acting β_2_-agonists (LABA) can directly act on the airway by administration as inhalable liquid or dry powder preparations, which have contributed to obvious benefits in the clinical treatment of asthma. However, this treatment can not affect underlying immunologic dissonance triggered by allergy.

Compared to conventional medication therapy, allergen immunotherapy (AIT) can generate long-term therapeutic effects even after the cessation of therapy on account of its influence on the allergic process and the establishment of immune tolerance, and is considered the only aetiology-based treatment for allergy diseases (5). The mechanisms of immune tolerance induced by AIT include but not are not limited to the induction of regulatory T (Treg), suppression of allergy-specific T helper (Th) 2 cells and upregulation of IgG produced by B cells (6). AIT can reduce medication use, improve lung function and asthma-related quality of life in asthmatic children (7). Furthermore, subcutaneous immunotherapy (SCIT) has been found to decrease allergen-specific AHR (8). In view of the favorable clinical benefits of AIT, more research needs to be carried out to support and guide the clinical applications.

T lymphocyte-driven inflammation is considered the principal pathophysiological mechanism of asthma (9). Recent studies have suggested an imbalance between Th17 and Treg cell in asthmatics which may trigger the progress of asthma (10, 11). Our previous study showed that the imbalance of Th17/Treg was associated with AHR in asthmatic patients (12). However, few studies have examined the effects of asthma treatments on the Th17/Treg balance. Thus, we aimed to further investigate the effects of ICS/LABA and SCIT on the Th17/Treg balance, to explore the mechanism of asthma treatments and further expand the understanding of the role of the Th17/Treg balance in asthma.

## Methods

### Study Subjects

In this study, 45 children aged 5-12 years old were enrolled, including 15 healthy children and 30 children with moderate to severe asthma who needed inhaled ICS/LABA treatment. The diagnosis of asthma and the corresponding asthma pharmacologic treatment were based on the criteria of the Global Initiative for Asthma (GINA). All asthmatic children were confirmed to be allergic to dust mites and had tested positive for house dust mite (HDM)-specific IgE (sIgE). Glucocorticoids or immunotherapy had not been administered to these patients within the month prior to inclusion in the study. All subjects had no other chronic diseases, nor were they using any long-term medication, and the healthy children had no allergic diseases, including asthma, rhinitis and food allergy. Subjects had no acute infections during the week prior to study enrolment. The study was approved by the Ethical Committee of the Second Affiliated Hospital and Yuying Children’s Hospital of Wenzhou Medical University in China.

**Figure 1:**
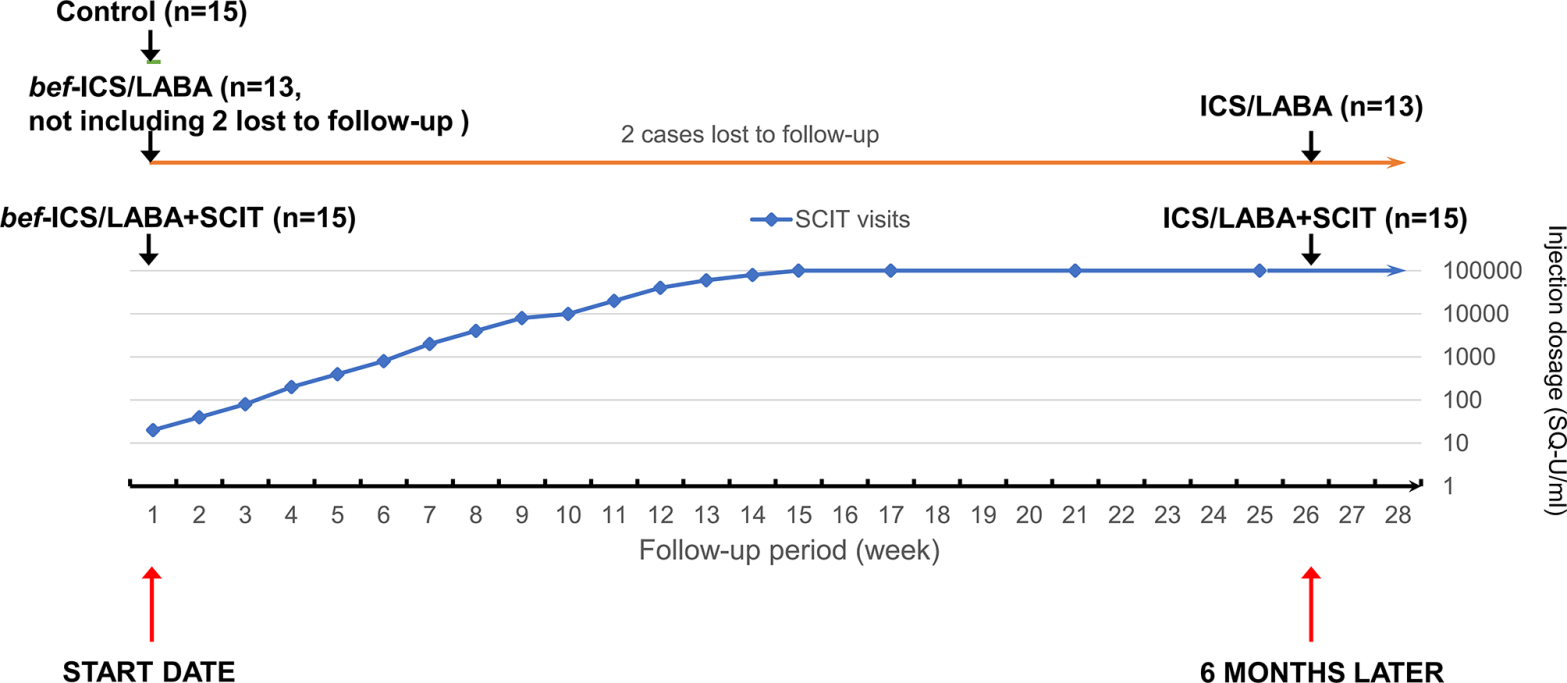
The Flow chart of dosing schedule of SCIT injection and grouping follow-up.

Among these asthmatic children, 15 asthmatic children were enrolled in the ICS/LABA groups treated with ICS/LABA therapy for 6 months, while the other 15 asthmatic children in the ICS/LABA+SCIT group were administrated combination therapy involving both ICS/LABA therapy and HDM-subcutaneous immunotherapy for 6 months. Budesonide and Formoterol Powder for Inhalation (AstraZeneca AB, UK) was adopted for ICS/LABA therapy. The dosage was customized by the specialist according to the condition of each patient. The patients undergoing HDM-subcutaneous immunotherapy received regular subcutaneous mite allergen injections (Alutard-SQ, ALK-Abelló, Denmark) for 6 months. For the first 15 weeks, the patients were injected once a week, and the injection dosage was gradually increased from 100 to 10000 SQ-U/ml. Afterwards, the patients were injected at a dosage of 10000 SQ-U/ml every four weeks. Peripheral blood samples were collected when the subjects were enrolled, and the subjects who received treatment were taken again after 6 months. *bef*-ICS/LABA group and *bef*-ICS/LABA+SCIT group respectively represent the two treatment groups before corresponding treatments. The Flow chart of dosing schedule of SCIT injection and grouping follow-up was showed in **Figure 1**.

### HDM-Specific IgE Measure

In measuring serum HDM-sIgE, the Pharmacia UniCap assay (Pharmacia Diagnostics, Uppsala, Sweden) was used to obtain standardized and comparable data. Patients were considered not allergic to dust mites when serum HDM-sIgE levels were determined to be in the range of 0 kua/l to 0.35 kua/l, defined as grade 0. HDM-sIgE levels higher than 0.35 kua/l are defined as an allergy to dust mites. The positive sIgE value is divided into 6 grades, from 1 to 6, which are ≥0.35 kua/l and <0.70 kua/l, ≥0.7 kua/l and <3.5 kua/l, ≥3.5 kua/l and <17.5 kua/l, ≥17.5 kua/l and <50.0 kua/l, ≥50 kua/l and <100 kua/l, ≥100 kua/l, respectively.

### Enzyme-Linked Immunosorbent Assay (ELISA)

Peripheral blood samples from subjects were collected in heparin anticoagulant tubes. The concentrations of IL-17A and IL-10 in the isolated serum were determined using ELISA (Boyun Biotechnology Ltd., China) according to the manufacturer’s protocols.

### Flow Cytometric Analysis

The peripheral blood mononuclear cells (PBMCs) were isolated from peripheral blood by human peripheral blood lymphocyte separation fluid (TBD Science, China) within 4 hours after sampling. For CD4^+^IL-17A^+^ Th17 cell analysis, the isolated PBMCs were cultured for 5 hours in the presence of PMA/Ionomycin/BFA/Monensin Mixture (Multi Science, China). The cultured PBMCs were stained with FITC-labelled CD4 antibody. Then, the PBMCs were processed with a Fix and Perm cell permeabilization kit (Multi Science, China) for intracellular staining. Then, the processed PBMCs were stained with a PE-labelled IL-17A antibody. For CD4^+^CD25^+^CD127^low^ Treg cell analysis, the isolated PBMCs were targeted with FITC-labelled CD4, APC-labeled CD25 and PE-labelled CD127 antibodies. Matching fluorescence-conjugated antibodies were adopted for preparation of the isotype controls. The antibodies used were purchased from BD Bioscience, USA. The stained cell suspension was analyzed by FC500 flow cytometer (Beckman Coulter, USA) and FlowJo V10 Software (TreeStar Inc., OR).

### Statistical Analysis

Statistical analyses were performed using GraphPad Prism 7 (Pearson’s GraphPad Software, Inc., USA). The data was displayed as mean ± SEM. Chi-square test, one-way ANOVA and t-tests were used to analyze differences between groups. A *P* value of less than 0.01 was considered statistically significant.

## Results

### Characteristics of the Study Population

In this study, 45 children were enrolled, including 15 healthy children and 30 asthmatic children. Moreover, 15 asthmatics were administrated by ICS/LABA treatment and the other 15 asthmatics were administered ICS/LABA treatment combined with SCIT treatment. However, 2 cases of asthmatics treated with ICS/LABA were lost to follow-up due to the change of living place and could not come to our hospital for follow-up, which were not included in the statistics. According to **Table 1**, there were no significant differences in age or gender composition among these three groups. There were no significant differences in SIgE grade, eosinophils, PEF%pred, FEV_1_%pred and FEV_1_/FVC between the ICS/LABA group and the ICS/LABA+SCIT group (**Table 1**).

**Table 1:** Characteristic of the study population.

	Control	ICS/LABA	ICS/LABA+SCIT	*P*
N	15	13	15	
Age (years)	9 (7,11)^a^	7 (6,9.5)	8 (6,10)	NS
Sex (male/female)	9/6	7/6	10/5	NS
Constitute (moderate/severe asthma)		11/2	12/3	NS^b^
ICS initial dosage (μg/day)		184.6 ± 16.66	192 ± 17.1	NS^b^
SIgE grade (0/1/2/3/4/5/6)	15/0/0/0/0/0	0/0/1/2/2/5/3	0/0/1/0/2/4/8	NS^b^
Eosinophils (%)		5.169 ± 1.077	7.453 ± 0.864	NS^b^
Eosinophil count (×10^9^/L)		0.449 ± 0.099	0.680 ± 0.072	NS^b^
PEF%pred (%)	94.07 ± 3.055	74.65 ± 3.673	80.69 ± 4.565	NS^b^, <0.01^c^, <0.01^d^
FEV_1_%pred (%)	106 ± 3.068	86.28 ± 3.735	87.24 ± 3.669	NS^b^, <0.01^c^, <0.01^d^
FEV_1_/FVC (%)	86.85 ± 1.179	89.46 ± 1.483	88.63 ± 1.971	NS

a Median (25% Percentile,75% Percentile).

b ICS/LABA v.s. ICS/LABA+SCIT.

c Control v.s. ICS/LABA.

d Control v.s. ICS/LABA+SCIT.

NS, no significance.

### Th17/Treg Imbalance in Asthmatic Subjects

**Figure 2:**
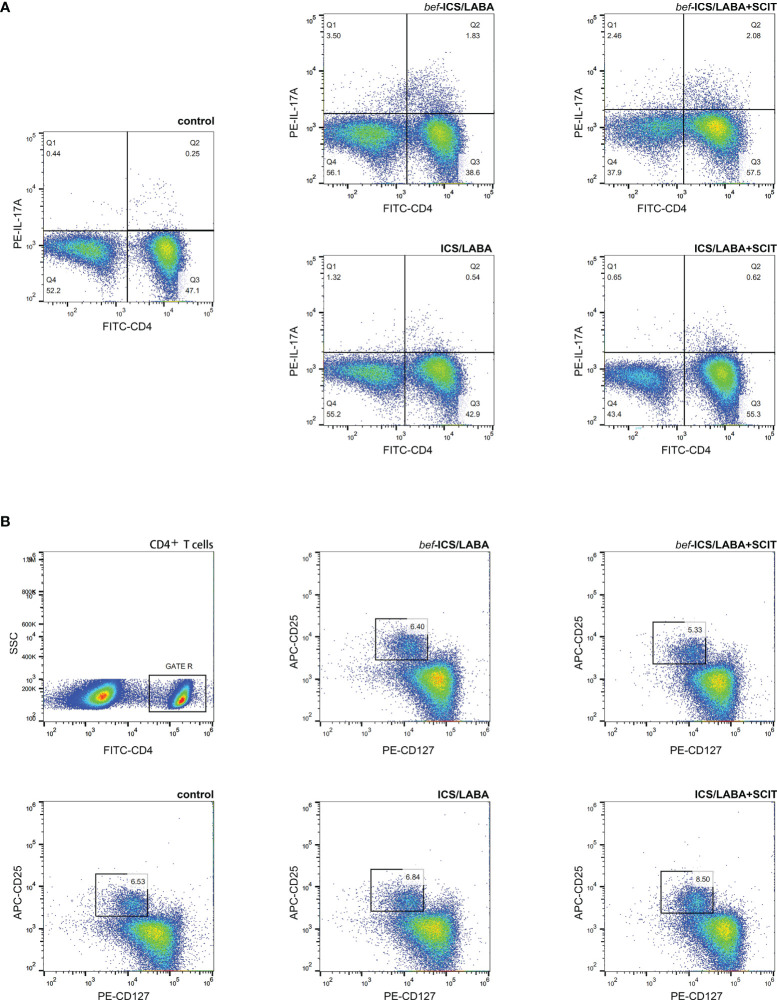
Representative flow cytometric analysis figures of CD4^+^IL-17A^+^ Th17 cells **(A)** and CD4^+^CD25^+^CD127^low^ Treg cells **(B)**.

**Figure 3:**
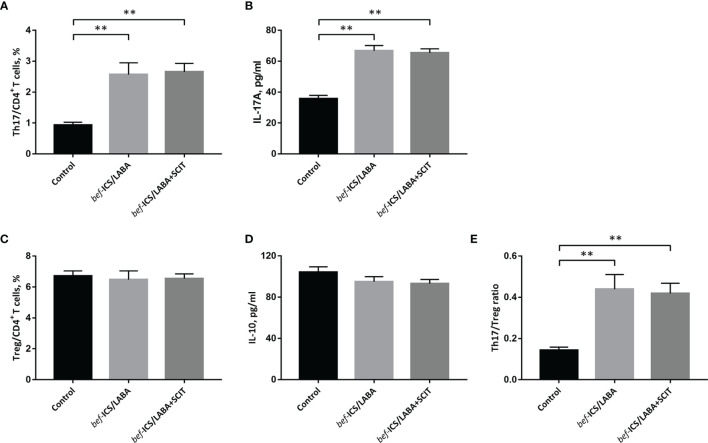
The percentage of Th17 cells in CD4^+^ T cells **(A)**, IL-17A **(B)**, the percentage of Treg cells in CD4^+^ T cells **(B)**, IL-10 **(D)** and the Th17/Treg ratio **(E)** from individuals in each groupThe monitoring parameters at the time of subject enrolled were compared between groups. **(A)** The percentage of Th17 cells in CD4^+^ T cells, **(B)** IL-17A, **(C)** the percentage of Treg cells in CD4^+^ T cells, **(D)** IL-10, **(E)** the Th17/Treg ratio. Statistical analyses were performed by unpaired *t* test for unpaired comparison. ^**^P < 0.01.

We measured the proportions of Th17 and Treg cells in peripheral blood by flow cytometry (**Figure 2**), as well as associated cytokines by ELISA. According to **Figure 3A**, the *bef*-ICS/LABA group (2.567 ± 0.383%, *P*<0.01) and *bef*-ICS/LABA+SCIT group (2.654 ± 0.273%, *P*<0.01) showed higher Th17 proportions than the control group (0.932 ± 0.093%). Similarly, the levels of IL-17 in serum in the *bef*-ICS/LABA group (66.75 ± 3.442 pg/ml, *P*<0.01) and *bef*-ICS/LABA+SCIT group (65.41 ± 2.616 pg/ml, *P*<0.01) were significantly higher than those in the control group (35.81 ± 2.088 pg/ml), respectively (**Figure 3B**). However, the proportions of Treg and IL-10 levels showed no significant difference among these three groups (**Figures 3C, D**). It is worth noting that the Th17/Treg ratios in the *bef*-ICS/LABA group (0.439 ± 0.072, *P*<0.01) and *bef*-ICS/LABA+SCIT group (0.419 ± 0.049, *P*<0.01) were higher than that in the control group (0.143 ± 0.015), suggesting a Th17/Treg imbalance in asthmatic subjects (**Figure 3E**). In addition, these indicators showed no significant difference between the *bef*-ICS/LABA group and the *bef*-ICS/LABA+SCIT group (**Figure 3**).

### Effect of ICS/LABA on Th17/Treg Balance

**Figure 4:**
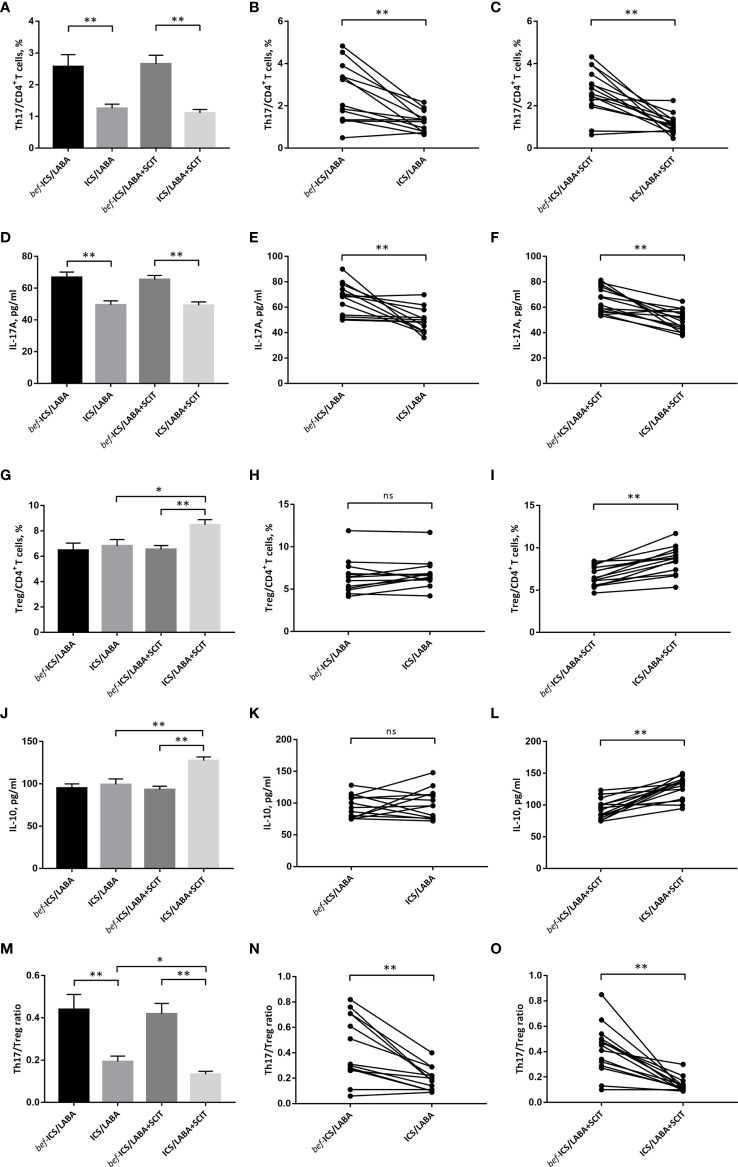
The percentage of Th17 cells in CD4^+^ T cells **(A-C)**, IL-17A **(D–F)**, the percentage of Treg cells in CD4^+^ T cells **(G-I)**, IL-10 **(J–L)** and the Th17/Treg ratio **(M-O)** before and after treatment. Connecting lines indicates data obtained in individual before and after corresponding treatment. Statistical analyses were performed by paired *t* test for paired comparison (*bef*-treatment vs. treatment patients), while unpaired *t* test for unpaired comparison (ICS/LABA treated vs. ICS/LABA+SCIT treated control subjects). ^**^P < 0.01, ^*^P < 0.05, ns, no significance.

After ICS/LABA treatment, the proportion of Th17 cells in peripheral blood showed a significant decrease (1.254 ± 0.134% vs. 2.567 ± 0.383%, *P*<0.01) (**Figures 4A, B**). Similarly, the level of IL-17 in serum was significantly decreased after ICS/LABA treatment (49.42 ± 2.643 pg/ml vs. 66.75 ± 3.442 pg/ml, *P*<0.01) (**Figures 4D, E**). However, ICS/LABA treatment did not affect the Treg proportion or IL-10 level (**Figures 4G, H, J, K**). Finally, the results showed a decrease in the Th17/Treg ratio after ICS/LABA treatment (0.194 ± 0.025 vs. 0.439 ± 0.072, *P*<0.01) (**Figures 4M, N**).

### Effect of ICS/LABA+SCIT on Th17/Treg Balance

There were significant decreases in the proportion of Th17 cells (1.109 ± 0.114% vs. 2.654 ± 0.273%, *P*<0.01) and IL-17 levels (49.23 ± 2.131pg/ml vs. 65.41 ± 2.616pg/ml, *P*<0.01) in peripheral blood after ICS/LABA+SCIT treatment (**Figures 4A, C, D, F**). Unlike single ICS/LABA treatment, ICS/LABA+SCIT treatment improved the proportion of Tregs (8.483 ± 0.408% vs. 6.549 ± 0.299%, *P*<0.01) and the level of IL-10 (127.4 ± 4.423 pg/ml vs. 93.15 ± 4.046 pg/ml, *P*<0.01) (**Figures 4G, I, J, L**). Furthermore, ICS/LABA+SCIT treatment significantly reduced the Th17/Treg ratio (0.133 ± 0.015 vs. 0.419 ± 0.049, *P*<0.01) (**Figures 4M, O**).

### Major Effect of SCIT on Th17/Treg Balance

There was no significant difference in the effects of ICS/LABA treatment and ICS/LABA+SCIT treatment on the Th17 proportion and IL-17 level (**Figures 4A, D**). However, the proportion of Tregs (8.483 ± 0.408% vs. 6.833 ± 0.485%, *P*<0.05) and the level of IL-10 (127.4 ± 4.423 pg/ml vs. 99.34 ± 6.496 pg/ml, *P*<0.01) in the ICS/LABA+SCIT group were significantly higher than those in the ICS/LABA group (**Figures 4G, J**). Correspondingly, the Th17/Treg ratio in the ICS/LABA+SCIT group (0.133 ± 0.015) was significantly lower than that in the ICS/LABA group (0.194 ± 0.025) (*P*<0.01) (**Figures 4M**). These data suggest that SCIT might mainly affect the Treg proportion and thus the Th17/Treg ratio.

## Discussion

A growing number of children suffer from asthma and it has been reported that a large proportion of asthmatics are attributable to allergy (13). To date, there has been a relatively complete set of medications for asthma to improve clinical symptoms. AIT has been used clinically for many years to treat allergic asthma from the etiology. In our study, children with moderate to severe asthma were administrated ICS/LABA treatment, some of whom were also treated with SCIT. Then, we found that circulating Th17 cells, IL-17A levels and the Th17/Treg ratio of asthmatic children were reduced after ICS/LABA treatment. ICS/LABA combined with SCIT treatment not only reduced the Th17 proportion and IL-17 levels, but also increased the Treg proportion and IL-10 levels, thus significantly reducing the Th17/Treg ratio.

T cell-driven inflammatory processes are thought to be involved in asthma. In exploring the role of T cells in protective and pathogenic responses in asthma, the classical Th1/Th2 paradigm provides a simple framework to reflect the balance of proinflammatory cells and immunosuppressive cells in asthma (14). In recent years, many studies have shown that Th17 cells and Th17 secreted cytokines such as IL-17A are involved in inflammation in asthma and promote the development of asthma. Treg cells are thought to be involved in immune tolerance and are derived from the same precursor cells as Th17 (15). Th17 and Treg cells are mutually antagonized during differentiation (16), but their interaction does not simply manifest as one waning and the other waxing. Therefore, the Th17/Treg paradigm has been proposed to improve the theory of proinflammatory and immunosuppressive balance in asthma. Many studies have confirmed the existence of Th17/Treg imbalance in asthmatic individuals (10, 11). Furthermore, Th17/Treg imbalance is supposed to be associated with AHR in asthma (12). In this study, we explored the Th17/Treg imbalance in asthma treatment aiming to better understand the asthma treatment mechanism and expand asthma combined therapy.

GCs are the most commonly used drug for asthma treatment. A previous study had found that oral GCs could decrease airway IL-17 in moderate-to-severe asthmatics (17). *In vitro* research showed that methylprednisolone could downregulate IL-17A levels in the supernatants of PBMCs from children with moderate and severe asthma (18). Fatemeh F et al. found that ICS treatment could decrease the number of IL-17^+^ cells in bronchial submucosa from both atopic and nonatopic asthmatic patients (19). Another study had found that IL-17A and Th17 cells in the plasma of patients with moderate asthma could be suppressed by inhaled budesonide and formoterol treatment (20). Similarly, inhaled ICS/LABA decreased Th17 inflammation in children with moderate to severe asthma according to our study. A lot of evidences indicate that GCs can ameliorate Th17 inflammation in asthma, but the effect of GCs on Treg has not been clearly determined. Some earlier studies have shown that GCs can increase and activate CD4^+^CD25^+^ Treg cells in asthmatic subjects (21–23). However, as the understand of Treg cell deepens, CD4^+^CD25^+^ T cells are not considered to be Treg cells entirely. In allergic mice model, GCs decreased the proportion of CD4^+^CD25^+^Foxp3^+^ Treg cells in the BALF and lung digests (24). Another study showed that the proportion of CD4^+^CD25^+^Foxp3^+^ Treg cells in the peripheral blood of asthmatic patients was not significantly elevated after inhaled budesonide and formoterol treatment for 12weeks (20). *In vitro*, budesonide and formoterol did not affect the CD4^+^CD25^high^CD127^-^ Treg population in cultured PBMCs (25). In this study, ICS/LABA treatment did not significantly increase Treg proportion *in vivo*. In summary, our study suggested that ICS/LABA treatment ameliorated Th17/Treg imbalance in asthma mainly by reducing Th17 inflammation.

AIT relieves allergic symptoms and prevents future allergen sensitizations by inducing long-lasting tolerance to the allergen (26, 27). The upregulation of Treg cells is an essential component of the immunological mechanisms of AIT-induced immunologic tolerance (6). A sustained Treg response is associated with the effectiveness of AIT (28). Very few reports are available to explain the effect of AIT on Th17 inflammation. Oral food-allergen immunotherapy was reported to decrease Th17 frequencies in patients aged 10 and over but not in patients under 10 years old (29). Man T et al. found that sublingual immunotherapy could decrease Th17 cells in the peripheral blood of children with allergic asthma. However, Nieminen K et al. reported that sublingual immunotherapy could not reduce allergen-stimulated IL-17A secretion in allergic patients (30). In our study, ICS/LABA combined with SCIT treatment significantly reduced Th17 response, but had no significant advantage over single ICS/LABA treatment. We considered that the initial six month SCIT had far less effect on Th17 inflammation compared to ICS so that the effect of SCIT on Th17 might be masked. In addition, our study showed that Treg was significantly increased in the initial stage of SCIT, and the combined of SCIT treatment was more effective in reversing Th17/Treg imbalance. The results suggested that SCIT firstly affected the Treg-induced immune tolerance mechanism rather than directly inhibited Th17 inflammation. In the initial stage of SCIT, the combined use of ICS/LABA was benefit to reverse proinflammatory and immunosuppressive imbalance on account of its inhibition to inflammation.

ICS treatment is the mainstay of anti-inflammatory pharmacotherapy for asthma, in connection with their capacity to target inflammatory cells. AIT is recommended as a supplementary therapy for asthma (31), because it mediates immunosuppression and induces a long-lasting immunological tolerance to allergens. We found that the combination of ICS treatment and AIT might be more conducive to the establishment of immune balance. The limitations of this research should be considered. First, this was a prospective study without randomization. Owing to SCIT costing extra hospital visiting time and expenditure, whether to use SCIT treatment depended on the patients. Second, there were no asthmatics administrated by SCIT treatment alone, as this could not be implemented in patients with moderate to severe asthma. We could only conclude that SCIT cannot significantly enhance the inhibitory effect of ICS/LABA on Th17 inflammation, but it did not fully indicate that SCIT had no effect on Th17 inflammation. Third, the clinical outcomes have not been monitored to explore the relationship between clinical benefits of ICS/LABA and SCIT with the balance of Th17 and Treg.

## Conclusion

ICS/LABA and AIT act on the proinflammatory and immuno-suppressive parts of the Th17/Treg balance, respectively. The addition of SCIT treatment enhanced the reversal effect of ICS/LABA on Th17/Treg imbalance in asthma.

## Data Availability Statement

The original contributions presented in the study are included in the article/supplementary material. Further inquiries can be directed to the corresponding authors.

## Ethics Statement

The studies involving human participants were reviewed and approved by the Ethical Committee of the Second Affiliated Hospital and Yuying Children’s Hospital of Wenzhou Medical University in China. Written informed consent to participate in this study was provided by the participants’ legal guardian/next of kin.

## Author Contributions

HD and RZ conceived the study. HD, RZ, and WZhang were responsible for patient recruitment and follow-up. LW, JW, and YT carried out the experiments and analyzed the results. RZ contributed to the writing of the manuscript. WZhang and WZhao contributed to the proofreading and revise. All authors reviewed the manuscript and approved the final manuscript as submitted.

## Funding

This work was financially supported by the Zhejiang Provincial Health Science and Technology Major Projects (WKJ-ZJ-2133) and the National Natural Science Foundation of China (81770030).

## Conflict of Interest

The authors declare that the research was conducted in the absence of any commercial or financial relationships that could be construed as a potential conflict of interest.

## Publisher’s Note

All claims expressed in this article are solely those of the authors and do not necessarily represent those of their affiliated organizations, or those of the publisher, the editors and the reviewers. Any product that may be evaluated in this article, or claim that may be made by its manufacturer, is not guaranteed or endorsed by the publisher.
